# How to Report Anecdotal Observations? A New Approach Based on a Lesson From “Puffin Tool Use”

**DOI:** 10.3389/fpsyg.2020.555487

**Published:** 2020-10-20

**Authors:** Krisztina Sándor, Ádám Miklósi

**Affiliations:** ^1^Department of Limnology, University of Pannonia, Veszprém, Hungary; ^2^MTA-PE Evolutionary Ecology Research Group, University of Pannonia, Veszprém, Hungary; ^3^Department of Ethology, Eötvös Loránd University, Budapest, Hungary; ^4^MTA-ELTE Comparative Ethology Research Group, Budapest, Hungary

**Keywords:** tool use, puffin, anecdotes, case study, scratching

## Background

There has been a long history of anecdotal reports in the field of natural history and comparative (evolutionary) animal behavior. Although, at the time of writing there is an open call for researchers of animal behavior by one of the oldest journal of the field “BEHAVIOR” to report “anecdotal evidence of unique behavior” (Kret and Roth, 2020), nowadays we see a decreasing trend of reporting anecdotes in scientific journals (Ramsay and Teichroeb, 2019). We do not dispute the relevance of publishing rare and novel behaviors or events, as they can be important drivers for future research, but we would like to draw attention to the fact that these reports should follow some standards and authors should be careful in avoiding over-interpretations.

An example of possible over-interpretation is a recently published article (Fayet et al., 2020a) that also received a lot of media hype (e.g., 79 news outlets at the time of writing; for more details see Altmetric, 2020). The authors reported on two separate occasions (one accidental field observation and one recorded on an 11 s long video) when two individuals of the Atlantic puffin (*Fratercula arctica)* were seen picking up or holding a stick in their beak, which then touched their body. These two cases were reported as an “*Evidence of tool use in a seabird”* (Fayet et al., 2020a).

This publication was followed by at least three commentaries [Auersperg et al., 2020; Farrar, 2020; von Bayern et al., 2020; and for further discussion see also Recommendation of the Farrar (2020) commentary by Dechaume-Moncharmont (2020)] that provided partly supportive or alternative views on the original report. The present authors share some of the doubts presented earlier but in this contribution we use the above case as an example to point out the problems with such anecdotal observations in general, and suggest ways to improve the information exchange among researchers.

## What Is an Anecdote?

In the study of animal behavior an anecdote may be referred to as providing an account of a rare behavior or event that has been observed either once or few times (Sarringhaus et al., 2005). The most critical factor to rely on this form of communication is the rarity of this observation in comparison to other cases. It may be important to note that the word “anecdote” is also an anthropomorphism because the typical definition of the anecdote in humanities describes this form of storytelling as recalling an event with some moral lesson often in a humorous style.

However, if the key feature of the observation is its rarity then it could be defined also as an anomaly, that is, something unexpected and/or different from typical situations. Note that this difference is not only playing with words. Reporting an anomaly would lessen the burden of the observer to present an “explanation” (“the lesson of the story”). In addition, it underlines the need to establish a baseline for comparison to the “typical” situation. Most anecdotes report cases when the animal's behavior implies some specific and/or complex new feature based on the assumption that this habit is advantageous (in terms of fitness) both for the individual and the species. However, one should also consider that an anomaly could also be disadvantageous, and may represent a malfunctioning.

However, we also noted that “anomaly” has a negative connotation in many fields of biology and medicine, thus we suggest to refer to *behavioral rarity* instead of an anecdote or anomaly (see below). We are aware that the term “anecdote” has a long history in animal behavior but terminological clarification can often lead to improvements in research quality. The new term expresses both the rare or single occurrence of the observation but at the same time refers to this event as having a particular value (for future research).

Some authors also refer to such reports as “anecdotal evidence” (Kret and Roth, 2020). One may ask what does “evidence” mean in this context? Something may be either “anecdotal” or evidence based. In the case of the former the report is based on a subjective account provided by the observer (but see issues on the accuracy of witness reports, e.g., Itsukushima et al., 2002). In the latter case the observation may be supported by data that do not represent evidence *per se* but have evidential value in relation to some claims, and that are as much as possible independent from identity of the observer. Such additional information may come from pictures, videos etc. published databases or papers from other researchers. Today it may be a minimum condition to provide video data for all reported anecdotes but even in such cases one may refrain from using the word “evidence” (see below).

With these general considerations about anecdotal reports in mind, in the following section we present an analysis and critical evaluation of the 11 s long observation on one puffin, provided by Fayet et al. (2020a).

## Describing Behavior

In case of reporting observations on animal behavior the accurate definition and description is a must. Unfortunately, this is often a weak point in many studies in which the authors do not provide adequate definitions, and the detailed description of the behavior is also missing.

In the original publication Fayet et al. (2020a) write “*adult puffin picks up a wooden stick from the ground then uses it to scratch its chest feathers*.” Watching the same video Auersperg et al. (2020) indicate that the bird “*simply accidentally touched its plumage with the stick while bringing it toward its breast*.” In their reply the authors (Fayet et al., 2020b) extend their description by adding”…*Its head stops in time such that the stick neither bumps against the body nor shifts/dislodges from the beak, and upon contact the head moves side to side in a scratching motion*.”

The basic problem here is mixing up the levels of analysis. Auersperg et al. (2020) use descriptive terminology, while Fayet et al. (2020b) refer to the action sequence as if it had some function (“scratching”). Importantly, there is general consensus that scratching has some function, e.g., by removing parasites (Bush and Clayton, 2018). However, for an objective analysis of any behavior the former is the preferred approach, especially if there are uncertainties about the interpretation. Also, note that Fayet et al. fail to provide a definition of scratching in the original paper and also in their responses (Farrar, 2020; Fayet et al., 2020b).

Indeed, we could not find good definitions and descriptions of similar actions for puffins and other bird species in the published literature. Thus, we decided to look through more than 40 videos (available on youtube) to identify action sequences that appear to be similar and might reflect “scratching” in various bird species. Generally, this analysis led us to conclude that (1) scratching (as distinguished from preening) consists of many usually a very rapid, repeated (stereotyped) movements (<1 s long), (2) it aims at a specific anatomical region of the body, (3) the body part (leg or beak) or the tool used for the action exerts some force on the skin surface (Auersperg et al., 2020).

In addition, we also reviewed numerous videos on scratching and preening behavior in Atlantic puffins, and then analyzed 7 videos frame by frame (for more details, see ESM, Part I and Supplementary Table 1, Sándor, 2020a). Based on these analyses, we concluded that puffins use their feet to repeatedly rub (scratch) parts of their bodies which they do not reach with their beak (e.g., head, neck), by moving it very rapidly in close contact with their body surface.

In all other cases, their beaks were used in two different ways: (1) they immersed their beak deep into the feathers and made small sidewise or up and down movements, and (2) they dragged the breast feathers one or more times between the mandibles of the beak (preening). We conclude that all of the analyzed actions were in all important aspects very different from that reported by Fayet et al. (2020a, for more details, see ESM, Part I), as “scratching.”

While looking for the videos for the above analysis, we also noticed that puffins shake their heads regularly and it is not uncommon for them to do so when they are holding nest material in their beak. Therefore, we also analyzed 7 videos in which puffins shake their heads with and without nest material in their beak. After a detailed description of their action sequences (see ESM, Part II and Supplementary Table 2, Sándor, 2020b), we conclude that these actions are more similar to the video published by Fayet et al. (2020a) than scratching or preening behaviors.

Even this short behavioral analysis provided important data for the possible interpretation of the behavior. Note that in the ESM we also reveal some data that picking up sticks is actually not so rare in puffins (for a similar argument see Dechaume-Moncharmont, 2020) thus the observed behavior could be also related to some nest building activity.

## Causal Interpretations

In their response to Farrar (2020), the authors indicate that it is unlikely that “*two cases we observed in puffins occurred by chance*.” Unfortunately, this remark represents a typical fallacy that gives strong arguments to those who object to publication of such records. The observation that on weekends about 40 people win some kind of lottery in the European countries does not mean that they can foretell the future. Importantly, the authors may be right (or wrong) but this is a dead end for the debate. Auersperg et al. (2020), Farrar (2020), and the present authors find it just as likely that the witnessed observations is the outcome of chance events. Accordingly, an alternative explanation is that the bird picked up a stick and then suddenly shook the head or tried to scratch (or preened) its feathers (see above) but the stick in its beak actually hindered the execution of the action. Furthermore, note that both observations on puffins were interpreted as providing some benefit for the actor. Just for the sake of it, why is it less likely that both birds suffered from a “stick-picking syndrome?” This could be a malformation of the behavior when for some presently unknown reason, some puffins cannot stop taking regularly sticks into their beaks. Although this idea may sound odd, so far nobody proposed any argument that could exclude it.

The claim about tool use is a very critical one because Fayet et al. (2020a) notes that this observation may indicate seabirds “*possessing sophisticated cognitive abilities*.” However, it is not clear what they mean by this hypothesis. First, it is not supported by the behavioral analysis (see above), second, interactions with objects do not need to be controlled by complex cognitive processes. Although, we agree with Dechaume-Moncharmont (2020) that reports on observations of animal behavior should not become the “victim of Morgan's canon,” alternative (simpler) explanations should be given similar attention. Cognitive abilities, as any other traits, should be subjected to a thorough experimental analyis before serious claims can be made. If the presently reported behavior was a combination of nest-building and scratching actions then there is no need to assume any sophisticated cognitive skill.

## Evolutionary Interpretations

An important point of Fayet et al. (2020a) report is to understand the evolution of the cognitive aspects of behavior. However, rare and likely accidental behaviors, like this one displayed by the puffin, do not need evolutionary explanations until their function and potential benefit has not been established (see above).

Since novelty is a relative term, we would be very reluctant to describe such an example as novel based on a single (or two) observation. The significant aspect of the observed behavior, picking up an elongated object, is very likely part of nest building behavior in puffins that has been probably subject of stabilizing selection. Accordingly, it is normal or expected that puffins pick up objects accidentally (see also Auersperg et al., 2020), and they may become part of other on-going activities, especially before or after the main nesting season. So having more time to observe puffins' behavior, we may find other instances of odd interactions with various types of objects but this does not confirm the presence of specific selective (evolutionary) processes.

## Behavioral Rarity as a Result of Environmental Effects

The role of the rearing and living environment should also be taken seriously because it may contribute to the emergence of various behaviors. However, it is a realistic possibility that such a specific or even seemingly novel trait is fully dependent on the environment, that is, there is no genetic variation present, so its role in the evolution of the species is questionable.

Keas may provide an example for this case. They are apparently very skillful tool users in captivity (e.g., Auersperg et al., 2010) but only very few such observations have been observed in nature (Auersperg et al., 2015, but see Goodman et al., 2018, for rare but systematic observation on kea tool use in the wild). One explanation could be that in keas tool use may emerge under very specific conditions spontaneously, and that it is not influenced by genetic variation.

Although parrots are often credited with being efficient tool users (Auersperg et al., 2018), the overall majority of such observations were done on captive individuals. Interestingly, Fayet et al. (2020a) also mention that parrot may use objects for scratching their head or back. However, videos available suggest that scratching with objects is actually very different from scratching with leg in parrots. The former action is carried out much slower and is reminiscent to kind of self-massage. Until this behavior is not observed in nature and without further detailed analysis, parrot tool using for scratching their body should not amount to claims that this behavior is part of the typical natural ethogram. Thus, what we see on these videos is not an evolutionary significant invention but a spontaneous manifestation of a behavior under specific condition by a few individuals.

We should acknowledge that the specific facilitative role of the environment can be excluded in the puffins' case because sticks are probably typical features of their habitat.

## Behavioral Rarity as a Result of Developmental Noise

The concept of developmental noise has been introduced to explain phenotypic variation with individuals of the same genotype based on stochastic processes at the molecular level (e.g., Willmore and Hallgrímsson, 2005). Specific traits (extremes) emerging as a result of such variation are not inherited and thus do not contribute to the evolution of the species. Such individuals may be re-occurring in the population but their rate depends solely on the actual environment and the trait cannot be selected for. Linneweber et al. (2020) have provided a convincing case that due to non-heritable asymmetry of some special neuron cluster in the *Drosophila* brain, some individuals perform much better in tasks requiring visual orientation. They argue that intrinsically stochastic processes lead to some individuals with “outstanding” skills that are stable over their life time. Obviously, one can claim that such skills are still within the capacity of the *Drosophila* species but this variation does not contribute to their evolution.

Thus, based on the above argument, both puffins in the report could represent such rare (“anomalous”) individuals (see also above) that may be specially interested in interacting with sticks, and eventually “get scratched/touched” by them. It follows, however, then in this case they should display this activity regularly.

## From Behavioral Rarities to Case Studies

Research practices change over time, and what seemed to be useful many years ago, might not provide benefits in the future. With the advent of (video) camera traps, we see a large number of videos showing so far unreported and unlikely sequences of interactions between animals within and between various species. Many of such observations could be important for generating new hypotheses (Dechaume-Moncharmont, 2020), however, before attributing evolutionary significance to them, one has to confirm the prevalence of the behavior within and between individuals, and also aim to take control over it (observe it) under more controlled conditions. Note that thousands of hours of behavior recordings increase the chance to observe any kind of rarities (anomalies). Thus, the reluctance of journals to publish such reports is understandable (Ramsay and Teichroeb, 2019).

However, there are ways to solve this issue by moving from anecdotes to behavioral rarities (again, the change in the label would point to a more objective position) and even to report so-called case studies which include a more objective methodology and analysis. We also suggest that reporting behavioral rarities by providing pure descriptions without any further analysis of available data should not be regarded as publications.

In contrast, case studies represent a widely accepted method for scientific investigations, in medicine, sociology etc. and over the years this type of research underwent methodological reforms (e.g., Levy, 2008; Thomas, 2011). Thus we encourage the research community of animal behavior to aim for publishing case studies rather than behavioral rarities (formally known as anecdotes).

## Toward Standardization of Case Studies in Animal Behavior

In order to facilitate information exchange among researchers, avoid or minimize misunderstandings and unnecessary debates, we make a first attempt to provide a non-exhaustive check list of aspects that should be considered for the preparation of a publication on behavioral rarities. Note that to some extent these points follow Tinbergen's 4 questions, and many of them have been suggested by various scholars earlier. We only made a compilation of these insights:

Behavioral analysis (accompanied by video recordings)

- Describe the observed actions/interactions in detail: What is the animal doing with its leg, body, head etc?
- Define the behavior and if any function is implicated, indicate the supposed benefit: What is the most appropriate label for this sequence of actions?
- Review the relevant literature on the target species or related species and provide a comparative analysis at the behavioral level (or give a statement that there exists no data to the best knowledge of the authors).

Functional analysis and hypotheses

- What is the possibility that the behavior occurred by chance? Provide estimates of baseline frequency of similar behaviors and actions (data from other species could be also informative)
- Consider the benefit of the action for the individual, and compare it with alternative behaviors that may lead to similar advantageous outcomes
- Discuss the possible disadvantages of this behavior or the possibility of behavioral malformation
- Provide long-term or repeated observations on a single individual
- Search for similar reports attributing function in the same or other species (from the wild or captivity).

Cognitive analysis and hypotheses

- Consider whether the observation is detailed and precise enough to make any assumptions on the underlying mental processes
- Argue why a more complex hypothesis on the mental processes has a higher probability to explain the observed behavior, and provide alternative explanations
- Suggest (and execute) possible ways (experiments) to test the phenomenon under more controlled laboratory conditions or by some planned manipulation at the site of the observation
- Consider whether particular aspects of the development (e.g., captivity) may make this observation special.

Evolutionary considerations

- Based on the above answers, discuss critically whether the observed behavior had or has the potential to play a role in the evolution of the species
- What particular aspects of the development (e.g., captivity) may make this observation special?

## Author Contributions

ÁM and KS conceived the idea of this opinion together and wrote the text. All authors contributed to the article and approved the submitted version.

## Conflict of Interest

The authors declare that the research was conducted in the absence of any commercial or financial relationships that could be construed as a potential conflict of interest.
