# Supplementary material for: How to Report Anecdotal Observations? A New Approach Based on a Lesson From “Puffin Tool Use”
Source: Front Psychol. 2020 Oct 20;11:555487. doi: 10.3389/fpsyg.2020.555487 (PMC7606989; doi:10.3389/fpsyg.2020.555487)
Supplement: Supplementary file 1 [file Data_Sheet_1.docx]

**Electronic supplementary material**

Title: **How to report anecdotal observations? A new approach based on a lesson from 'puffin tool use'**

Authors:

Krisztina Sándor, Department of Limnology, University of Pannonia; Veszprém, Hungary;

MTA-PE Evolutionary Ecology Research Group, University of Pannonia, Veszprém, Hungary

and

Ádám Miklósi, Department of Ethology, Eötvös Loránd University, Budapest, Hungary

*Part I: Video analysis on scratching, preening and “tool use” of puffins*

The authors reviewed numerous videos on scratching and preening puffins, and then chose three scratching and three preening videos to analyze it in detail (Sándor, 2020a), then compare it to that reported by (Fayet et al., 2020a).

Based on these analyses **(Table S1)**, we concluded that puffins use their feet to repeatedly rub (scratch) parts of their bodies which they do not reach with their beak (e.g. head, neck). In all other cases, their beaks are used in two different ways: i.) they put their beak deep into the feathers and make small movements, and ii.) they drag the breast feathers one or more times between the mandibles of the beak (preening).

According to Fayet et al (2020a), the bird scratches with a stick on their video, but it is clearly seen that the stick hardly touches the surface of the feathers. As one can judge from the videos this bird does not make as forceful movements as a puffin does when scratching its feet, nor immerses the tip of the stick as deep into the feathers as when preening with its beak).

Thus we doubt we see an effective scratching on the video reported by Fayet et al (2020a). In our opinion, the possibility of preening can also be rejected, as the bird’s action do not lead to zipping its feathers or spreading uropygial gland oil or removing parasites from it. Furthermore, the puffin touches a part of its body with the stick that is also easily reachable with the beak, so it can either be preened or scratched more efficiently with the beak (Auersperg et al., 2020; Farrar, 2020).

Thus we conclude that all of the analysed actions were in all important aspects very different from that reported by Fayet et al (2020a, for more details).

*Part 2: Video analysis on head shaking during manipulation of nest material*

Although the authors rejected the possibility that the bird is taking up nest material, we also examined how puffins behave during nest material collection. Watching a few freely available videos (see below), we found that puffins shake their heads regularly (Sándor, 2020b), and this is often done when holding nest material in their beak (this may reflect antiparasitic behavior, or attempt to discard objects from the beak; **Table S2**).

We argue that the puffins’ behaviour in these videos is more similar to the video published by Fayet et al. (2020a) than actions described as scratching or preening. Although Fayet et al. (2020a) state that puffins preferentially collect soft nest material in their studied colonies, the possibility of occasionally gathering sticks for this purpose cannot be completely rejected either because there are reports of this happening (Short, 2011; RSPB, 2017; Charman, 2018).

They also argue that *„videos recorded after the tool-use episode showed the stick on the ground, confirming that the bird did not take it to its nest. We are therefore confident that our observed puffins did not pick up the sticks as nest-lining material*”. Furthermore, they also mentioned that “*our observations were made during chick rearing, beyond the peak of courtship and nest making*.” (Fayet et al., 2020b).

We do not agree with this conclusion, because (1) although the video was not taken at the peak of the breeding season, the observed bird may have been a late nester (we do not get detailed information about this from their article) and (2) it is also possible that birds pick up the stick for nest repair. Thus, the fact that the bird on the video attempted to collect nest material cannot be rejected out with absolute certainty. One possible explanation for why the bird did not take the stick to its nest is that after picking up the stick the bird “realized” that the object was not suitable for nest material. Thus, the video actually reveals the moment when the bird is trying to discard the picked stick from its beak (by slight head shaking), and by accident the object touches its belly.

**Table S1**. The results of the frame by frame video analysis of scratching, preening (Videos 1 to 6) and, the reported behaviour by Fayet et al. (2020a; video 7) of Atlantic puffins.

**Table S2.** The results of the frame by frame video analysis of head shaking (Videos 1 to 6) and the reported behaviour by Fayet et al. (2020a; video 7) of Atlantic puffins.

**Links to videos used for the video analyses:**

**1^st^ compilation (Sándor, 2020a):**

1. <https://www.youtube.com/watch?v=bT4W4GtsP_g> (from 1:22)
2. <https://www.youtube.com/watch?v=Y3Fw8kYc0Es&t=14s> (from 0:09)
3. <https://www.youtube.com/watch?v=oCDnfJBYAkc> (from 1:29)
4. <https://www.youtube.com/watch?v=yrhRYANapDo&t=184s> (from 1:10)
5. <https://www.youtube.com/watch?v=POYjjqPhbjE> (from 1:01)
6. <https://www.youtube.com/watch?v=0a2hmxzpDF8> (from 1:11)
7. <https://www.youtube.com/watch?v=1i7wFLF0UJE>

**2^nd^ compilation (Sándor, 2020b):**

1. <https://www.youtube.com/watch?v=whZQisSPs5U>  (from 0:18)
2. <https://www.youtube.com/watch?v=-3fuXwZWDS4> (0:12)
3. <https://www.youtube.com/watch?v=cspJ2rGAYRM>  (from 0:33)
4. <https://www.youtube.com/watch?v=sQznv-UM27U&t=37s> (from 1:16)
5. <https://www.youtube.com/watch?v=znksAQOXnww> (from 1:34)
6. <https://www.youtube.com/watch?v=vu5uVaepTn0&t=34s> (from 2:34)

**References:**

Auersperg, A. M. I., Schwing, R., Mioduszewska, B., O’Hara, M., and Huber, L. (2020). Do puffins use tools? *Proc. Natl. Acad. Sci. U. S. A.* 117, 1073. doi:10.1073/pnas.2001988117.

Charman, B. (2018). A puffin moves nesting material in the rain-soaked landscape. *photograph*. Available at: https://www.bretcharmanphotography.com/brets-blog/skomer-island-puffins [Accessed July 17, 2020].

Farrar (2020). Evidence of tool use in a seabird? *Proc. Natl. Acad. Sci.* 117, 1277–1279. doi:10.31234/osf.io/463hk.

Fayet, A. L., Hansen, E. S., and Biro, D. (2020a). Evidence of tool use in a seabird. *Proc. Natl. Acad. Sci.* 117, 1277–1279. doi:10.1073/pnas.1918060117.

Fayet, A. L., Hansen, E. S., and Biro, D. (2020b). Reply to Auersperg et al.: Puffin tool use is no fluke. *Proc. Natl. Acad. Sci. U. S. A.* 117, 10–11. doi:10.1073/pnas.2003294117.

RSPB (2017). Puffin collecting nest material. *video*. Available at: https://www.youtube.com/watch?v=epyFiRgogYs [Accessed July 17, 2020].

Sándor, K. (2020a). A video collection to analyse the behavior of Atlantic puffins (*Fratercula arctica*). *video*. Available at: https://www.youtube.com/watch?v=xKmGbvpYcn4&feature=youtu.be [Accessed July 17, 2020].

Sándor, K. (2020b). A video collection to analyse the behavior of Atlantic puffins (*Fratercula arctica*) 2. *video*. Available at: https://www.youtube.com/watch?v=bpcesJivDvk&feature=youtu.be [Accessed July 17, 2020].

Short, J. (2011). A puffin carrying tree branches in its mouth. *photograph*. Available at: https://www.gettyimages.at/detail/foto/a-puffin-carrying-tree-branches-in-its-mouth-lizenzfreies-bild/126293941 [Accessed July 17, 2020].
